# Obesity and emergency care in the French CONSTANCES cohort

**DOI:** 10.1371/journal.pone.0194831

**Published:** 2018-03-26

**Authors:** Anne-Laure Feral-Pierssens, Claire Carette, Claire Rives-Lange, Joane Matta, Marcel Goldberg, Philippe Juvin, Marie Zins, Sebastien Czernichow

**Affiliations:** 1 Population-based Epidemiological Cohorts Unit, INSERM UMS 11, Villejuif, France; 2 Assistance Publique Hôpitaux de Paris, Emergency Department, Georges Pompidou european Hospital, Paris, France; 3 Assistance Publique Hôpitaux de Paris, Nutrition Department, Georges Pompidou european Hospital, Centre Spécialisé Obésité IdF, Paris, France; 4 Paris Descartes University, Paris, France; 5 Nutrition Department, Faculty of Agricultural and Food Sciences, Holy Spirit University, Jounieh, Lebanon; 6 INSERM, UMR 1153 Epidemiology and Biostatistics Sorbonne Paris Cité Center (CRESS), METHODS team, Paris, France; National Yang-Ming University, TAIWAN

## Abstract

**Context:**

Obese patients are raising specific questions in emergency care such as equipment issues or urgent procedures. Even though obesity prevalence and subsequent health expenditure are increasing worldwide, there is scarce literature about their specific resource utilization of Emergency Departments (ED). These few studies do not take into account both socio-economic situation and comorbidities which are well-known factors influencing healthcare use. Our objective was to assess the emergency care resource utilization of obese individuals (Body Mass Index (BMI) ≥ 30kg.m^-2^) compared to normal-weight individuals taking into account comorbidities and social-economic situations.

**Methods:**

The French CONSTANCES epidemiologic cohort is a randomly selected sample of French adults. Participants data are linked to the National Health Insurance Database collecting all medical acts. The rate of ED visits of obese (and each obesity class) and normal-weight participants were compared considering confounding factors (comorbidities, various socio-economic data). The primary endpoint was to have visited the ED between 2010 and 2013. Sex-separated analysis and multivariate logistic regression models were performed and adjusted odds-ratios [OR] (95% Confidence Intervals [CI]) were calculated.

**Results:**

We included 21,035 normal-weight and 5,003 obese participants. Obese participants visited the ED more often than normal-weight participants (men: 30.5% vs. 26.7%; women: 30.3% vs. 24.4%, *P*<0.001). Obese participants presented more comorbidities and a lower socio-economic situation than normal-weight participants. After adjustment, obese participants had a higher risk of visiting ED (men: OR = 1.18; 95% CI: 1.04–1.33; and women: OR = 1.36; 95% CI: 1.22–1.52), with a higher risk for class III participants (BMI ≥ 40 kg.m^-2^) (men: OR = 2.18; CI 95%: 1.32–3.63; and women: OR = 1.85; 95% CI: 1.38–2.49).

**Conclusion:**

Obese individuals have a higher level of emergency care resource utilization than normal-weight individuals and it increases with severe obesity. Further studies are needed to better understand their healthcare pathways leading to EDs.

## Introduction

Obesity is one of the leading causes of death and is related to a variety of comorbidities [1–4]. It has been shown to reduce life expectancy and is one of the main preventable causes of illness and premature death [1,5,6]. Its prevalence has been increasing over the last decades; in 2017, 12% of adults worldwide had a body mass index (BMI) ≥30 kg.m^-2^ and this prevalence has doubled since the 1980 [1]. A French cohort study estimated the prevalence of obesity among adults over 30 years old of 15.5% [7].

Beyond comorbidities, subsequent disabilities and their social consequences [8], obesity may result in up to 30% higher health expenditure than for non-obese patients [9–13]. However international comparisons are difficult because of differences between health care systems and access to care organization. Studies often estimate indirect health expenditure through weighted-expenditure of each disease related to obesity or through small size samples. Thus, plain amounts cannot be easily compared between healthcare systems [14] contrary to physicians visiting rates. While there is a broad medico-economic literature about obesity costs (mainly from US studies), there is scarce descriptions of visiting rates in different specialities or types of medical resources consumed and very little about emergency care [13].

Obese patients seem to visit the ED mainly for cardiovascular or respiratory complications [3,15,16]. Because its overall prevalence is increasing, there is likely to be more obese patients in the ED which raises specific questions and brings new challenges for healthcare delivery. Among them, urgent procedures could be more difficult to achieve (resuscitation maneuvers, intubation, drip insertion), clinical signs could be shaded, treatments’ dosages must be adapted correctly and imaging strategies are different (less relevance or success of ultrasonography) [16–20]. There are also logistical difficulties because of inadequate material (limited access to bariatric chairs or beds) and obese patients need more staff for nursing procedures which are longer than for normal-weight patients [21]. Finally, the complexity of these situations seems to increase with the obesity’s severity. Because of all the modifications and changes that taking care of obese patients imply, it seems important to assess in the obese population their use of emergency care in order to prepare healthcare providers, emergency departments and physicians to be involved in this particular population. Despite different studies estimating that obese patients had an increased mean number of visits in primary care, few studies focused on emergency care [22–26], and none of them took into account both comorbidities and socio-economic status in the analysis. Besides, the association between social deprivation and increased medical and emergency care use is well-known [27].

We hypothesize that obesity is independently associated with a higher rate of ED visits. We aim at estimating the medical burden of obesity in the emergency medicine field. Our goal is to assess and compare the rate of ED visits of the obese population vs normal-weight participants of the French CONSTANCES cohort from 2010 to 2013.

## Methods

The CONSTANCES cohort is intended to serve as an epidemiological research infrastructure with a focus on occupational and social factors, and on chronic diseases and aging [28,29]. CONSTANCES began inclusions in 2012 and was designed as a randomly selected sample of French adults aged 18–69 years at inclusion; 200,000 subjects were to be included over a five-year period. Randomly selected eligible persons receive at home an invitation to come to a Health Screening Center for a comprehensive health examination and fill a questionnaire [28]. The follow-up includes a yearly self-administered questionnaire, and a periodic visit to a health center. The collected data included social and demographic characteristics, socio-economic status, life events, behaviours, and occupational factors. Social and work-related events and health data (purchased medications, medical consultations, hospitalizations) were collected from the French National Health Insurance Database. The Insurance Database concerns most of the French insured population and is an exhaustive public collection of each medical act (medication, imaging, outpatient visit, inpatient admission) that led to any public financing (fully or partially). The Insurance Database also includes information concerning specific listed chronic diseases that lead to full public financing.

### Anthropometric data

During the physical examination in the health center, the weight is measured in kilograms (kg) with a scale. Height is established with a measuring rod without shoes. The Body-Mass Index (BMI) is calculated as BMI = weight (kg)/height (m^2^) and classification is made following the World Health Organization [6]; BMI<18.5: underweight, BMI = [18.5;25[: normal weight, BMI = [25;30[: overweight, BMI ≥ 30: obesity. Obesity has also been separated in three sub-categories depending on the BMI (kg/m^2^); BMI = [30;35[: class I, BMI = [35;40[: class II, BMI ≥ 40: class III. Data collection followed standardized protocols and its quality control was considered efficient [30].

### Health data

Health data are collected from different sources (questionnaires, medical examination, Insurance database) [31]. Upon inclusion, participants fill self-administered questionnaires including information on lifestyle, health, physical limitations, social and personal characteristics and lifetime job history. They also benefit from an extensive health examination (medical, paraclinic exams and blood tests). The participants’ personal and familial clinical history is recorded by a physician. Patients are considered as presenting cardiovascular disease risk when dyslipidemia, diabetes or hypertension was self-reported, diagnosed by a health center physician or notified in the insurance database. Smoking status is recorded at the inclusion. Cardiovascular disease history comprises history of stroke, ischemic cardiopathy events or cardiovascular diseases notification in questionnaires or insurance database. Cancer history is recorded through insurance database notification.

### Socioeconomic data

Social and demographic characteristics include occupational class (OC) (as the current one or the longest held one for retirees), educational level, household composition, and material living conditions. “Social difficulties” marker is a binary indicator considered positive if participants declare having difficulties to read, calculate or write, have no access to internet or met a social worker. Refraining from health care in the year previous the cohort inclusion is self-declared. We collected through the insurance database the type of public insurance of our participants and screened for Public Universal Medical Coverage which is attributed to socially deprived individuals with a low-income level.

### ED visiting rate

Healthcare utilization is recorded through the National Health Insurance database. Data are complete from January the 1^st^ 2010 to December the 31^st^ of 2013. These data are linked to CONSTANCES cohort data for each participant that gives his agreement [30]. All emergency visits have been recorded.

### Endpoint

Our goal is to assess and compare the ED visiting rate of obese and normal-weight participants of the French CONSTANCES cohort from 2010 to 2013. Our primary endpoint is the rate of ED visits, defined as the proportion of participants having visited the ED at least once over the studied period.

### Statistical analysis

Continuous data are expressed as means and standard deviations (SD) if normally distributed. Non-normally distributed variables are expressed as median [interquartile range IQR]. Categorical data are reported as numbers and percentages. We performed sex-separated comparisons between normal-weight and obese participants by using Student *t* test for continuous data and chi-square test for categorical data.

We performed multivariate logistic regression models of ED visiting rate adjusting for potential *a priori* selected confounding factors, namely: age, sex, comorbidities (cardiovascular disease history, cardiovascular disease risk factors, smoking status), socio-economic (occupational class, social difficulties, medical coverage) and geographic factors. We estimated adjusted odds ratio [OR] and their 95% confidence interval [CI] of emergency visiting rate of obese participants, the ED visiting rate of normal-weight participants being the reference.

We followed the STROBE recommendations for reporting observational cohort studies [32]. Analyses were performed using Stata version 14.0 (StatCorp Ltd, College Station, USA). The CONSTANCES study was approved by authorities regulating ethical data collection in France (CCTIRS: Comité Consultatif pour le Traitement des Informations Relatives à la Santé; CNIL-Commission Nationale Informatique et Liberté) and all participants signed an informed consent.

## Results

### Population

**Fig 1:**
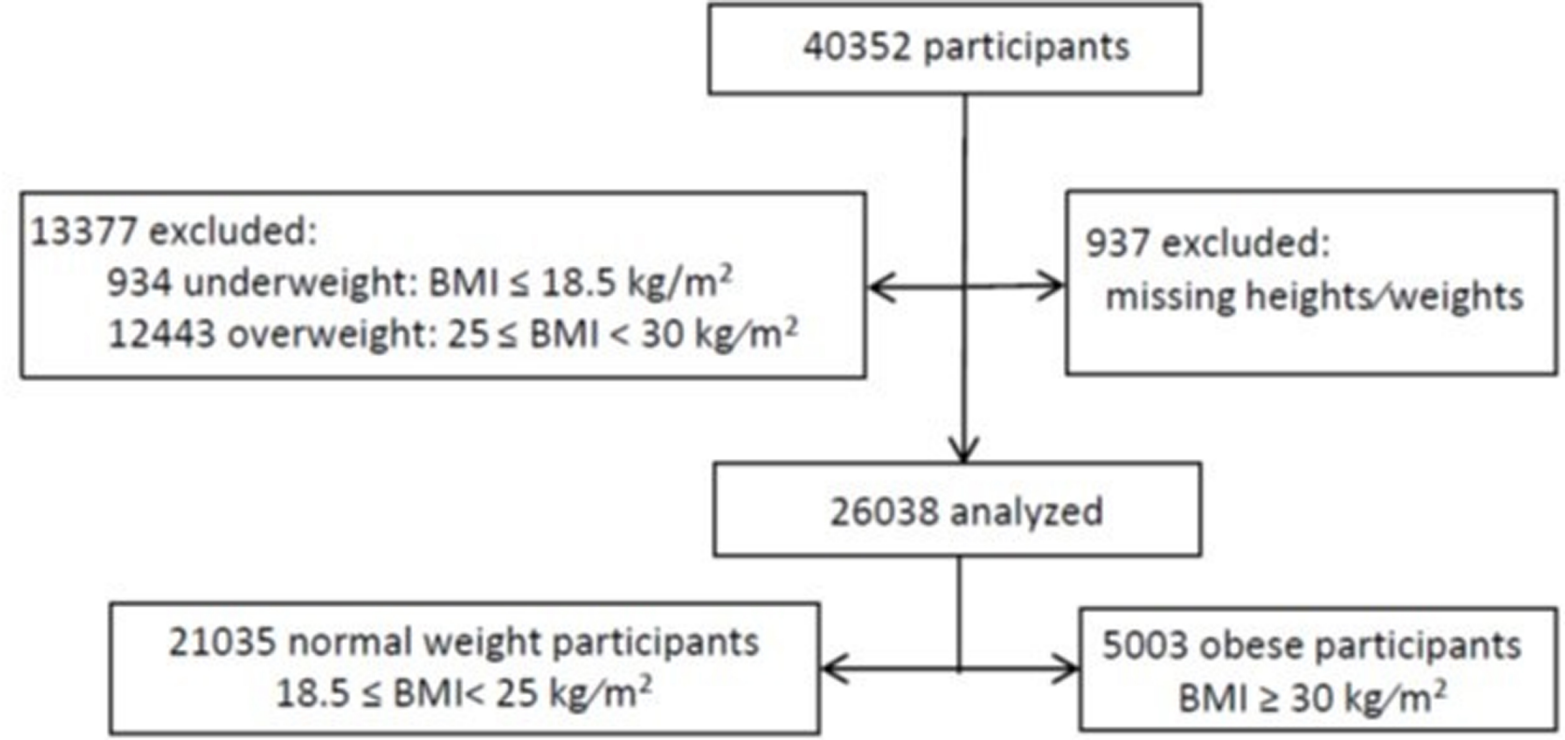
Flow diagram of participants, France, 2010–2013. BMI: Body Mass Index.

In September 2016, 40,352 participants with complete health care insurance data had been included in the CONSTANCES cohort. Some participants were excluded because of missing data on height or weight (n = 937); underweight and overweight participants were also excluded from the analyses (n = 13377). Our final sample included: 21,035 normal weight (8342 men and 12,693 women) and 5,003 obese participants (2,380 men and 2,623 women) (Fig 1).

### Comorbidities

Sex-separated analyses were performed. Obese participants were older than normal-weight participants for men (57(46.5,63.5) vs. 46(35.5,57.5) years old, *P* <0.001) and women (53(41.5,62) vs 47.5(36.5,58), *P* <0.001). At the time of their inclusion in the cohort, obese participants had different smoking behaviours than normal-weight participants. Among men, obese participants were mostly former smokers and less often current smokers than normal-weight participants. Among women, obese participants were more often non-smokers than normal-weight women. The prevalence of cardiovascular disease risk factors was substantial with 48% of obese men 38.1% of obese women. Cardiovascular disease history was more frequent in the obese than in the normal-weight population as for respiratory history (Table 1). Cancers were also more frequent for obese than normal-weight individuals (men: 6.2% vs 4.2%; women: 9% vs 7%, *P* <0.001). After adjusting for age, obese men had a higher risk than normal-weight men for cardiovascular disease risk factors (OR = 4.11; 95% CI: 3.68–4.59) and for cardiovascular events (OR = 2.07; 95% CI: 1.75–2.07). Same results were found for obese women for cardiovascular risk factors (OR = 3.46; 95% CI: 3.13–3.82) and for cardiovascular events (OR = 1.58; 95% CI: 1.29–1.95).

**Table 1:** Health, social and economic characteristics of obese (BMI≥30 kg/m^2^) vs. normal weight (18,5 kg/m^2^ ≤BMI<25 kg/m^2^) participants, France, 2010–2013.

	Men			Women		
	Normal N = 8342	Obese N = 2380	*P*-value	Normal N = 12693	Obese N = 2623	*P*-value
BMI, median(Q1,Q3), kg/m^2^	22.9 (21.6,24.0)	32 (30.9,34.2)		21.9 (20.5,23.3)	32.9 (31.2,35.8)	
Age, median(Q1,Q3), years	46 (35.5,57.5)	57 (46.5,63.5)	<0.001	47.5 (36.5,58.0)	53 (41.5,62.0)	<0.001
Smoker status, %			<0.001			<0.001
Non-smoker	43.2	30.2		49.8	55.4	
Current smoker	23.5	16.8		20.1	13.6	
Former smoker	33.3	53.1		30.1	31.1	
MD	4,8	6		4.9	5.9	
≥1 CV disease risk factor, %	13.2	48	<0.001	12.9	38.1	<0.001
Diabetes, %	1.7	12.8		2.7	11	
Hypertension, %	7.2	36.6		6.9	27.7	
Dyslipidemia, %	7.1	25.2		5.4	14.6	
MD	1.7	1.4		1.5	1.8	
History of CV disease^a^, %	2.7	9.9	<0.001	1.2	3	<0.001
MD	1.9	2.2		1.7	2.2	
History of respiratory disease^b^, %	9.1	9.6	0.427	8	13.1	<0.001
MD	2.8	3.2		2.5	3.7	
Self-reported dyspnea, %	10.2	36.5	<0.001	22.2	62.2	<0.001
MD	2.5	3.6		2.8	4.1	
Occupational class			<0.001			<0,001
Executives	40.4	27		25.1	12.7	
Employees	12.5	15		31.1	44.1	
Blue-collar workers	12.7	22		2.4	6.8	
MD	9.4	11.8		9.5	11.3	
Universal Medical Coverage	4	4.8	0.075	3	5.1	<0,001
Refraining from care in the last 12 months	11.3	16.7	<0.001	14.1	26.3	<0,001
Having social difficulties^c^	8.5	13.4	<0.001	8.7	15.1	<0,001
Social worker follow-up	2.7	5.2	<0.001	2.2	6.1	<0,001
Difficulties to read	4.5	7.2	<0.001	4.3	7.2	<0,001
Difficulties to write	2.6	4.8	<0.001	1.8	4.2	<0,001
Difficulties to count	2.7	3.6	0.317	3.4	5.5	<0,001
No access to internet	5.9	13.5	<0.001	5.2	12.6	<0,001
MD	1.2	2.4		1.1	1.5	

MD: missing data. CV: cardiovascular; Q1: first quartile; Q3: third quartile.

^a^Cardiovascular disease: myocardial infarction, stroke, ischemic cardiopathy.

^b^History of respiratory disease: asthma, COPD history.

^c^Having social difficulties: at least one among: difficulties to read, write or count, need of a social worker in the last year, no internet access.

### Socio-economic items

Occupational status distributions were different between obese and normal-weight groups. There were less executive among obese participants regardless of their sex. Obese men were more likely to be blue-collar (22%) than women who were more often employees (44.1%) (Table 1). Among obese women, 5.1% were subscribing to Public Universal Medical Coverage versus 3% of normal weight women (*P* <0.001). Almost half of the obese group declared having currently or recently financial difficulties to subsist (45.7% of obese men and 54% of obese women vs 29.5% and 35.5% respectively, *P* <0.001). Obese participants were more likely to meet with a social worker and to have social difficulties such as difficulties to write, to read or to calculate (Table 1). Among women, obese participants were twice as much as those with a normal weight to have a “social difficulties indicator” (15.1% vs 8.7%). Of note, 13.5% of obese men and 12.6% of obese women declared not having any access to internet (respectively 5.9% and 5.2% in normal-weight men and women, respectively).

### ED visiting rate

Obese participants did visit at least once the ED more often than normal-weight participants (30.5% of obese men vs 26.7%; 30.3% vs 24.4% in women, *P*<0.001). And this trend was more important in class III participants with 47.8% men and 39.6% of women visiting ED. Mean number of emergency visits were higher in obese than normal-weight participants and the difference is more important among women and in the class III category (Table 2).

**Table 2:** Emergency department visits for each obesity class participants and normal-weight participants from 2010 to 2013, France.

	Men				Women			
	Normal	Obese			Normal	Obese		
	N = 8342	Class I N = 1933	Class II N = 378	Class III N = 69	N = 12693	Classe I N = 1824	Class II N = 592	Class III N = 207
Proportion with ≥ 1 ED visit, %	26.7	30*	29.6	47.8*	24.4	29*	31.3*	39.6*
Mean ED visits, (SE)	0.41 (0.94)	0.47 (1)*	0.48 (1)	0.84 (1.5)*	0.37 (0.9)	0.52 (1.2)*	0.53 (1.1)*	0.78 (0.1)*
Mean ED visits if ≥ 1 visit, (SE)	1.5 (1.2)	1.6 (1.4)	1.6 (1.2)	1.8 (1.7)	1.5 (1.1)	1.8 (1.7)*	1.7 (1.3)	2 (1.7)*
Proportion of superusers^a^, %	0.2	0.2	0.5	1.5*	0.1	0.4*	0.3	1.5*

ED: Emergency Department; Nb: number; Q1: first quartile; Q3: third quartile. Normal-weight: 18.5 ≤BMI<25 kg/m^2^. Class I: 30 ≤ BMI < 35 kg/m^2^. Class II: 35 ≤ BMI < 40 kg/m^2^. Class III: ≥ 40 kg/m^2^.

^a^Superusers: participants with ≥ 8 visits over the period.

* *P-*value <0.05 when comparing each obesity class to normal-weight participants.

**Fig 2:**
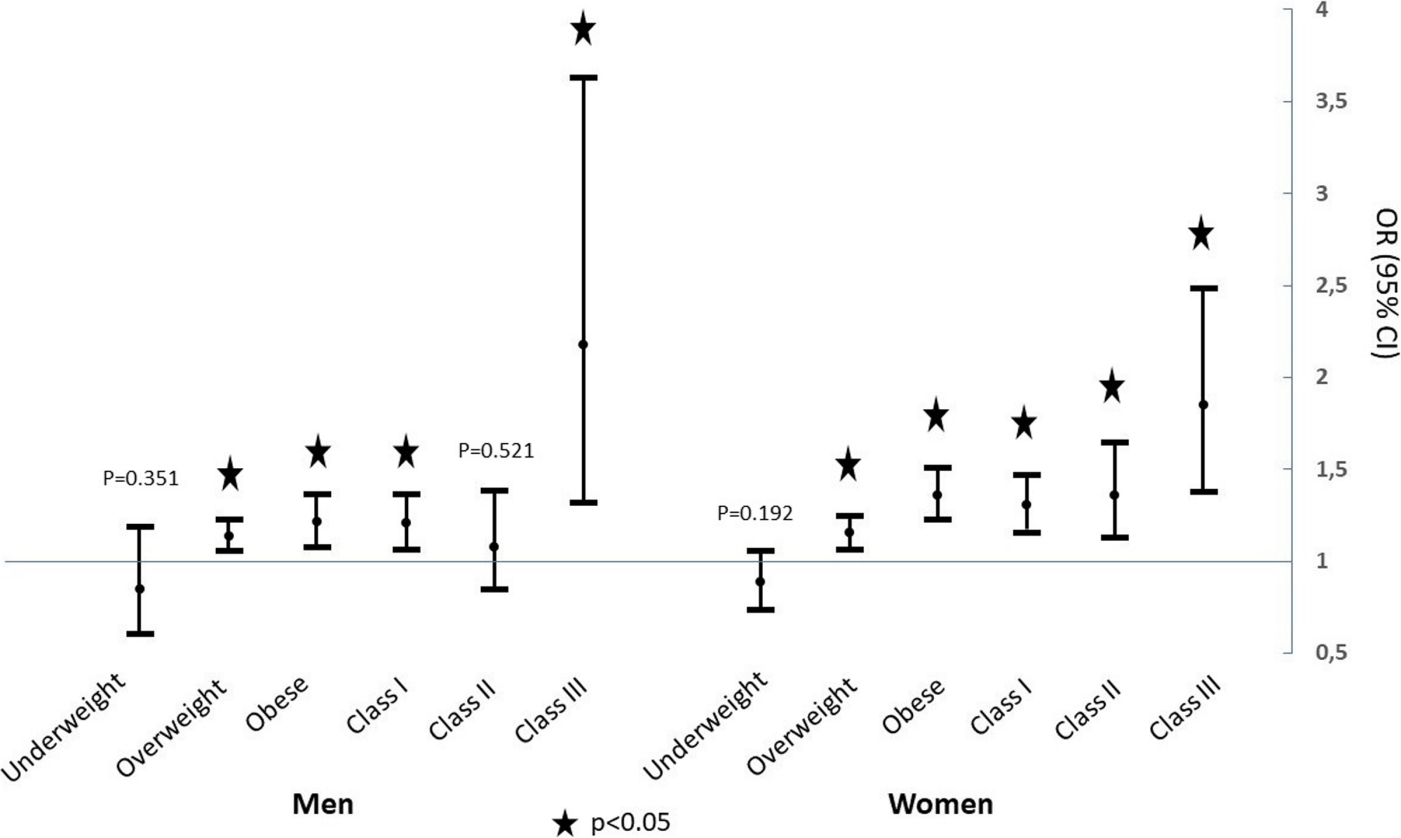
Multivariate odds-ratios (OR) of emergency visiting rate of each BMI class and obesity class participants compared to normal weight participants (95% confidence intervals). OR are adjusted on age, center, comorbidities, social difficulties and occupational class.

Multivariate logistic regressions adjusted showed that obese participants had a higher risk of visiting the Emergency Department compared to normal-weight participants. This result was even more striking among obese women (OR = 1.36; 95% CI: 1.22–1.52). However obese men had also a higher risk of visiting the ED, compared to normal-weight men (OR = 1.18; 95% CI: 1.04–1.33). The risk of visiting the ED was also higher in class III obesity: OR = 2.18; 95% CI: 1.32–3.63 for men and OR = 1.85; 95% CI: 1.38–2.49 for women (Fig 2).

## Discussion

Obese individuals have a higher risk of visiting ED compared to normal-weight individuals and these results are greater for women (OR = 1.36 for women vs 1.18 for men). It is even more important with class III obese participants.

Our results concerning higher emergency care utilization take into account factors usually associated with higher health care consumption such as age, comorbidities, cardiovascular factors or social deprivation [27]. Moreover, through our work we chose to adjust for various aspects of social deprivation. Our study confirms that obesity is linked to social and economic vulnerability. However, our work goes beyond usual indicators since we described precisely different aspects of social deprivation. We characterized details and nuances through financial difficulties notification, refraining from healthcare or social difficulties through reading or writing limitations.

Our results concerning the visiting rate of obese participants in the Emergency Department seems to confirm what has been described in other contexts. Obese individuals have a higher level of healthcare utilization [11,13,33,34]. A review from Kent et al. analysed 75 studies and concluded that obese individuals had a mean increase of annual healthcare costs of 36%, compared to normal-weight individuals. The mean increase concerning inpatient care costs was 34% and these results were higher for women than for men [9,13]. However, to our knowledge, there hasn’t been any study on ED care utilization among a broad obese population such as the study we conducted here. Bertakis et al. did not find any differences in ED utilization between obese and normal-weight population. On the contrary, Espallardo et al. found that ED utilization was higher for over class II obesity population but fail to take into account social variables throughout their analyses [25,34]. Other authors found a higher acute hospital admission rate and longer length-of-stay [26,33] but did not incorporate socio-economic status in the analysis. Moreover, social deprivation has been known to have multiple dimensions beyond occupational status or personal income and these dimensions are being addressed here [35]. The health status and comorbidities of our population are similar to what is usually described in other epidemiologic studies: obesity is associated to higher cardiovascular disease risk factors and respiratory disease history [1,36,37].

We observed that obese individuals (and class III especially) have a higher risk of visiting the ED. Other authors explained the higher level of annual costs attributed to obesity by the higher prevalence of cardiovascular diseases, osteoarthritis, cancers and other chronical diseases [34,36–38]. The recent review of Kent et al. showed that medication is the first reason for higher healthcare costs followed by inpatient and ambulatory care [13]. Some authors showed a higher primary care utilization among the global obese population. However, as we demonstrated here, this does not seem to narrow unplanned emergency care. Thus, our study is the first to understand and highlight specific higher ED visiting rate among class III obese individuals independently of age, comorbidities, health center and social conditions. This particular increasing population is associated with higher mortality and undergoes a major reduction in life expectancy compared with normal-weight population [39].

Class III individuals are associated with higher mortality and are undergoing a major reduction in life expectancy compared with normal-weight population [39]. This particular population has much more severe comorbidities and is more socially deprived than class I and II individuals. Moreover, severe obesity is associated with higher mobility limitations increasing difficulties in consulting or visiting physicians [40,41]. Transportation can be an issue and obese patients might delay seeking for medical advice when needed. Access to out-patient care can be weakened terminating in later emergency visit. Finally, individuals with severe obesity might have an increased blunt perception of their body compared to less severe obesity. Therefore, they can experience an erroneous and mitigated perception of their medical needs which can be another reason for delaying looking for medical care [42–44]. All these reasons could lead or participate to a higher risk of emergency visits as we assessed in our study. That is why our results should lead us to investigate in details primary care utilization among class III population in order to elaborate interventions and make health care pathways more efficient.

The main strengths of our study include its large sample size and its exhaustive database on Emergency Department visits and multi-dimensions socio-economic status. Moreover the anthropometric data were collected following standardized protocols which adds validity to our results [28]. However, our study has some limitations. Parts of socio-economic data have been collected through self-reporting. Self-administered questionnaire can lead to memory biases that could alter patient answers about social difficulties, recent financial limitation or refraining from care. In order to limit these consequences, we analysed other socio-economic indicators that are not influenced by personal interpretation as occupational status or benefiting from the low-income medical coverage and that were collected through national insurance database. Moreover, our primary descriptive analysis of socio-economic indicators shows higher social deprivation situations in obese than in normal-weight population which is consistent with the literature on this specific matter. Concerning comorbidities and medical history, these specific data have been collected through insurance database notification, self-reporting and physician assertion during medical examination in the health center. They are less subject to misinterpretation, under or over-reporting.

Respiratory disease history and dyspnea have been reported and included in Table 1 while describing our populations’ characteristics. As it has been described in literature, obese population seems to experience more often respiratory diseases than normal-weight population, this could lead to ED visits [3]. However, reported dyspnea is a symptom that did not seem to be linked to respiratory disease notification in the insurance database in our population. Chronic respiratory disease history was also self-reported and collected through insurance notification. But only severe respiratory disease could lead to notification, which is rarely done. Because of the lack of robustness of these two respiratory indicators, we decided not to implement them in our statistical model. Nonetheless, when implemented among other adjusting factors, the adjusted odds-ratio of ED visiting rate are still significant (results not shown).

At last, there could be a time lag between the data collected through insurance database and healthcare and socio-economic data collected upon inclusion in the cohort. Inclusions began in 2012 and insurance data were available and exhaustive from 2010 to 2013. Upon inclusion, patients could report a change in cardiovascular disease risk factors or socio-economic status that was not the same as during the period analysed through insurance database. That could have led to categorization errors.

## Conclusion

Our study shows that obesity is associated to different comorbidities and social deprivation factors which lead individuals to higher emergency care utilization. Our results report that obese patients have a higher risk of visiting ED than normal-weight patients. ED medical staff will be facing specific challenges with the increase in the obesity epidemic. Since ED are at crossroads between ambulatory or general practice and inpatient care, further studies concerning this population are needed to better understand their healthcare pathways leading to EDs, reasons to their visits and what happens downstream.
